# Identification of novel immune ferroptosis-related genes associated with clinical and prognostic features in breast cancer

**DOI:** 10.3389/fgene.2023.1173159

**Published:** 2023-04-12

**Authors:** Zhenlan Xie, Jialin Li, Chen Liu, Tie Zhao, Yixiang Xing

**Affiliations:** ^1^ Department of Pathology, Tongling People’s Hospital, Tongling, China; ^2^ Tongling Vocational and Technical College, Tongling, China

**Keywords:** immune, breast cancer, prognosis, biomarkers, ferroptosis

## Abstract

**Introduction:** Breast cancer is the most common form of cancer among women, it is critical to identify potential targets and prognostic biomarkers. Ferroptosis combined with immunity shows a pivotal role in a variety of tumors, which provides new opportunities to detect and treat breast cancer.

**Methods:** Our first step was to combine multiple datasets to search for immune ferroptosis-related mRNAs. In the next step, risk signatures were created using Least Absolute Shrinkage and Selection Operator (LASSO). After that, based on the results of the multivariate Cox analysis, we created a prognostic nomogram and validated the model’s accuracy. Finally, functional enrichment analysis, single sample gene set enrichment analysis (ssGSEA), immunity and drug sensitivity correlation analysis were performed to explore the possible mechanisms by which these immune ferroptosis associated mRNAs affect BRCA survival.

**Results:** An immune ferroptosis signature (IFRSig) consisting of 5 mRNAs was constructed and showed excellent predictability in the training and validation cohorts. A correlation analysis revealed that clinical characteristics were closely related to risk characteristics. Our nomogram model, which we created by combining risk characteristics and clinical parameters, was proven to be accurate at predicting BRCA prognosis. Further, we divided patients into lowrisk and high-risk groups based on the expression of the model-related genes. Compared with low-risk group, high-risk group showed lower levels of immune cell infiltration, immune-related functions, and immune checkpoints molecules, which may associate with the poor prognosis.

**Discussion:** The IFRSig could be used to predict overall survival (OS) and treatment response in BRCA patients and could be viewed as an independent prognostic factor. The findings in this study shed light on the role of immune ferroptosis in the progression of BRCA.

## 1 Introduction

With the highest incidence of all female malignant tumors worldwide, BRCA is the most prevalent malignant tumor in women (Siegel et al., 2021). Although great progress has been made in the therapeutic effect of BRCA (Siegel et al., 2023), sadly, there are still no reliable diagnostic tools or markers for determining the prognosis of BRCA patients (Islam et al., 2020; Pupa et al., 2021; Sindhu et al., 2021). Until now, tumor lymph node metastasis (TNM) stage has been used to predict BRCA prognosis and treatment response. However, due to tumor heterogeneity, BRCA patients with the same TNM stage showed different prognosis and treatment response. Therefore, it is important to combine other useful indicators to predict prognosis and treatment response.

As opposed to apoptosis, necrosis, and autophagy, ferroptosis was a type of programmed cell death dependent on iron (Hadian and Stockwell, 2020; Fardi et al., 2021; Jiang et al., 2021). The classical mode of regulation of ferroptosis was through the neutralization of lipid peroxides by glutathione peroxidase 4 (GPX-4) (Yang and Stockwell, 2016; Ding et al., 2020; He et al., 2020). There was growing evidence that ferroptosis causes hypersensitivity reactions in cancer cells with a higher degree of malignancy, particularly those with intrinsic or acquired drug resistance (Hangauer et al., 2017; Viswanathan et al., 2017). In addition, ferroptosis influences the effectiveness of cancer immunotherapy and was associated with T cell-mediated antitumor immunity (Wang et al., 2019). Additionally, it had been demonstrated that immune modulation of the tumor microenvironment (TME) could facilitate ferroptosis, which in turn increases the immunogenicity of the TME, enhancing the immune modulation response (Zhang et al., 2019). It was anticipated that immunotherapy will had synergistic effects through ferroptosis, promoting tumor control, in combination with ferroptosis-promoting modalities like radiation therapy and targeted therapy (Lang et al., 2019; Chen et al., 2021). There was a close relationship between tumor cells, the immune microenvironment, and ferroptosis (Lang et al., 2019; Jiang et al., 2021). In addition, studies had found that ferroptosis intervention could effectively improve immunosuppression (Gao et al., 2015; Sun et al., 2016; Alavian and Ghasemi, 2021). In conclusion, the important role of immunity and ferroptosis might provide a new direction for predicting prognosis and treatment response of breast cancer.

The goal of this research was to develop new survival predictive risk signatures and to explore the prognostic role of immune ferroptosis-related mRNAs in BRCA. Firstly, we combined multiple datasets to screen mRNAs associated with prognosis. The risk features for BRCA prognosis prediction were then constructed by LASSO regression analysis. At the same time, the total samples were divided into training cohort and validation cohort according to the ratio of 1: 1. Then, by combining this feature with other clinical parameters, a nomogram was created to predict 1-, 3-, and 5-year survival. Ultimately, we explored the relationship between risk characteristics and underlying biological function, immunity, and drug susceptibility.

## 2 Materials and methods

### 2.1 Transcriptome data acquisition and model building

In this research, we downloaded the transcriptome Fregments Per Kilobaseper Million (FPKM) of breast cancer patients from the TCGA database (https://portal.gdc.cancer.gov/). The RNAseq data in FPKM format was converted into transcripts per millionreads (TPM) format and log2 conversion was performed. Transcriptome data was organized and ENSG numbers were converted to symbolic IDs. The research was carried out in accordance with the Helsinki Declaration (revised 2013).

The ImmPort database (https://www.immport.org./home) and the GeneCard database (https://www.genecards.org/) were used to obtain 17,500 human immune-related genes (IRGs). A total of 398 ferroptosis-related genes (FRGs) were downloaded through the FerrDb database (http://www.datjar.com) and literature (Song et al., 2021). Two gene sets were crossed with differentially expressed genes to obtain co-expressed genes (IFR-DEG), and the cutoff conditions were set as log2 fold change (logFC) < 1, *p*-value <0.05. Then, univariate Cox regression analysis was performed, and the total samples were divided into training cohort and validation cohort according to the ratio of 1: 1. The training cohort builds a risk model based on LASSO-Cox regression analysis. The formula for calculating the risk score was as follows: Risk score = βgene1×exprgene1+βgene2×exprgene2+.+βgenen×exprgenen. At the same time, to reduce the dimensionality of the nomogram, we used an unsupervised learning algorithm called principal component analysis (PCA), which allowed us to visualize the spatial distribution of samples.

### 2.2 Gene correlation, gene network and functional enrichment analysis

Gene correlation analysis was performed by Spearman analysis and visualized with the ggplot2 package. Model-related genes were submitted through GeneMANIA (http://www.genemania.org), which analyzed and displayed genes that perform similar functions—representing protein expression and inheritance in the network. Genes were enriched by Gene Ontology (GO) terms and Kyoto Encyclopedia of Genes and Genomes (KEGG) pathways to investigate the potential biological functions of interacting proteins and model gene co-expression in pan-cancer, GO enrichment analysis including molecular function (MF), cellular component (CC) and biological process (BP). Both GO and KEGG analyses were performed by the R package ClusterProfiler. Then high and low risk differential genes were also analyzed by GO and KEGG.

### 2.3 Model validation

We divided patients into high-risk and low-risk groups, and then we generated heatmaps associated with prognosis based on the median risk score. To determine differences in survival between high- and low-risk groups, we plotted Kaplan-Meier survival curves, distributions of survival status, and distributions of risk scores. Finally, the predictions of the risk scoring model were further validated by applying the “timeROC” package to plot the ROC curves of the training and validation groups.

### 2.4 Independent prognostic analysis and nomogram construction

Nomograms were constructed by combining relevant clinical factors and risk scores obtained with risk scores (we used the R packages: “rms”, “foreign” and “survival”). An evaluation of the model’s discriminative ability was then carried out by drawing a calibration curve.

### 2.5 The relationship between risk score and immune cell infiltration

We calculated immune stages using single-sample gene set enrichment analysis (ssGSEA) (He et al., 2018). In exploring the relationship between risk score values and immune-infiltrating cells, we used Spearman’s rank correlation analysis.

### 2.6 Immune microenvironment, immune checkpoints, immune escape

A stromal score, an immune score, an estimated score, and a tumor purity were calculated using transcriptome profiles from UCECs. In the high-evolution and low-evolution groups of hub genes, we compared stroma scores, immune scores, estimated scores, and tumor purity using Limma and ggpubr packages. In addition, tumor immune escape mechanisms in different risk groups were analyzed using the TIDE algorithm.

### 2.7 Gene mutation analysis

In the gene mutation analysis, the number and quality of gene mutations in two subgroups (Maftools package) of BRCA patients were analyzed. In addition, we also analyzed the relationship between tumor mutational burden (TMB) and risk score subgroups using Student’s t-test.

### 2.8 Predicting response to chemotherapy

To elucidate the role of signatures in clinical treatment, IC50 values of commonly used chemotherapeutics were evaluated using high-throughput sequencing data of BRCA in TCGA. In this study, the Wilcoxon signed-rank test was used to compare the differences between the two groups, and pRRophetic and ggplot2 were used for the visualization of the results.

### 2.9 Statistical analysis

R software (version 4.1.2) was used for statistical analysis. For data processing, the Perl programming language is used. Prognostic significance was determined using multivariate Cox regression analysis. PCA was also performed using R’s ggplot2 package. The survival difference between the two groups was analyzed by Kaplan-Meier curve and logrank test was used. Gene correlations, risk scores and correlations between immune cells and immune genes were analyzed using Spearman’s correlation coefficient test. When *p* < 0.05, the difference was statistically significant.

## 3 Results

### 3.1 Construction of a prognostic risk model for differentially expressed genes related to immune ferroptosis

**FIGURE 1:**
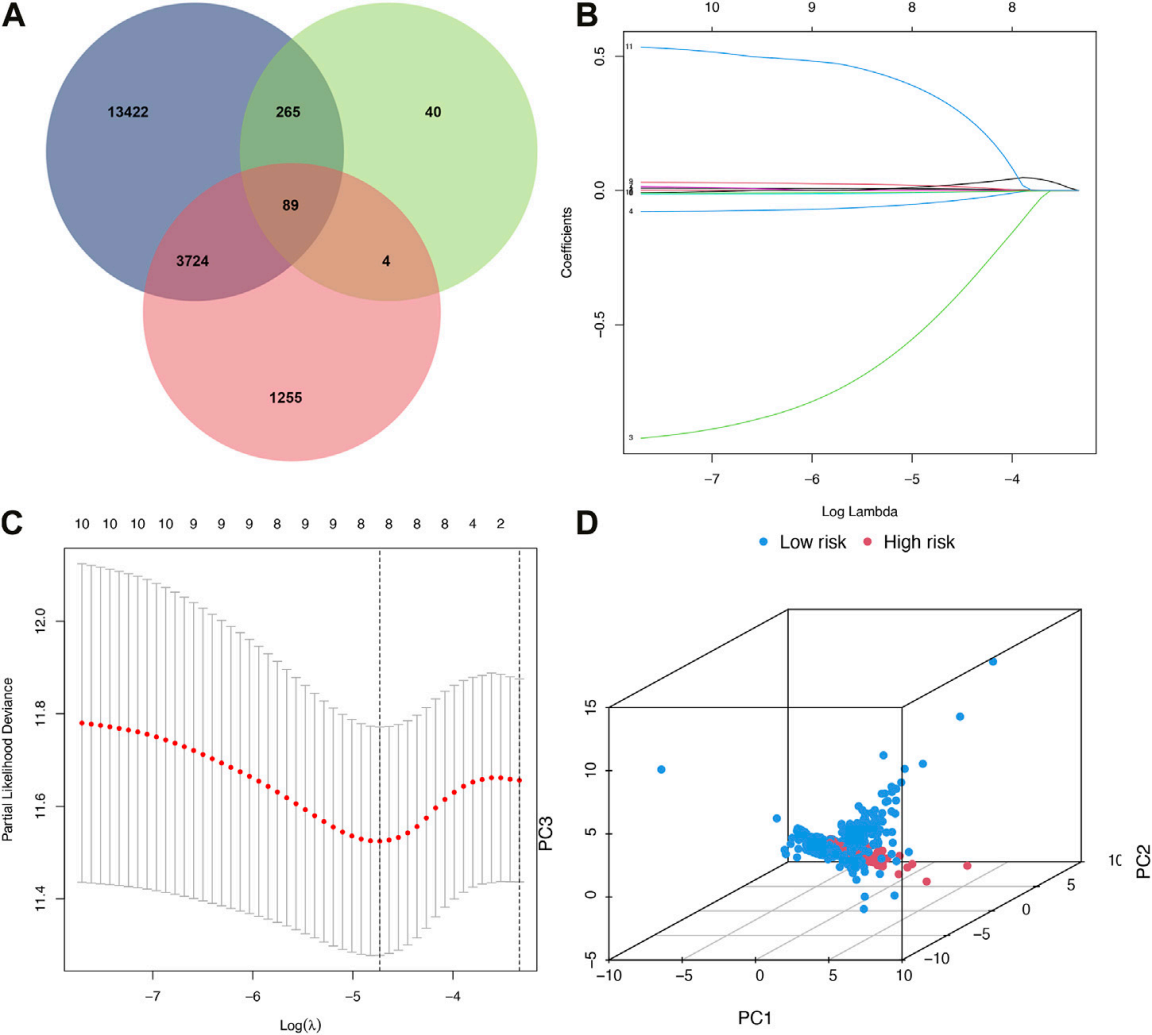
Build a risk model. **(A)** Venn diagram. Blue represented immunity genes, green represented ferroptosis genes, and red represented differential genes. **(B)** Distribution of LASSO regression coefficients for crossed genes. **(C)** LASSO deviation profile of crossed genes. **(D)** PCA plot of high and low-risk group. PCA, principal component analysis.

89 co-expressed genes were discovered by combining 17,500 immune-related genes, 398 ferroptosis-related genes, and 5072 BRCA differentially expressed genes (Figure 1A). A predictive model of immune ferroptosis-related risk was then constructed using lasso regression (Figure 1B). The risk score formula was: riskscore= (0.008*TFRC) + (−1.042*IFNG) + (−0.064*FLT3) + (−0.016*FZD7) + (−0.009 *SIAH2) (Figure 1C). Patients were divided into high- and low-risk groups based on the median risk score (50%). The results of PCA validated the differential expression of high- and low-risk groups in BRCA patients (Figure 1D).

### 3.2 Model gene correlation and functional enrichment analysis

**FIGURE 2:**
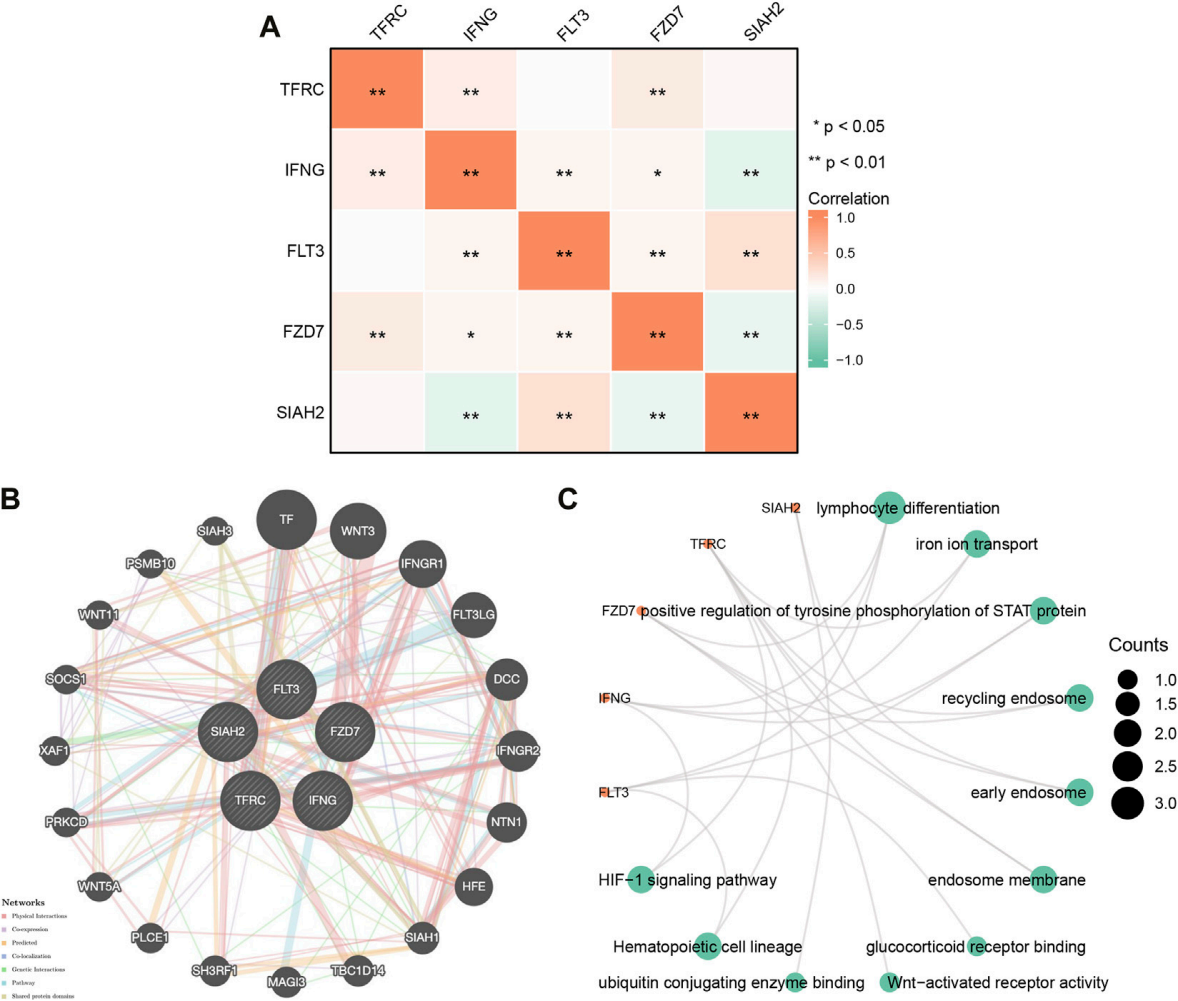
**(A)** Gene correlation network diagram of prognostic model. **(B)** Model -related gene network plotted using GeneMANIA. **(C)** Model gene enrichment analysis in pan-cancer: GO and KEGG.

To explore potential relationships of model genes, we examined correlations between Model-related genes using Spearman correlation analysis. As shown in Figure 2A, FLT3 negatively correlated with SIAH2, while TFRC positively correlated with IFNG and FZD7; SIAH2 positively correlated with FZD7 and SIAH2; TFRC positively correlated with FZD7 and SIAH2; FLT3 positively correlated with FZD7 and SIAH2; FZD7 was negatively correlated with SIAH2.

We constructed gene-gene networks through GeneMANIA to explore gene interactions. Figure 2B shows 20 nodes around the central node of the Model-related genes, which were genes related to the genes model based on physical interactions, co-expression, predictions, co-localization, genetic interactions, pathways, and shared domains. Among them, TF, WNT3, IFNGR1, and FLT3LG were ranked in the top. Regarding model-related genes GO and KEGG enrichment analysis, as shown in Figure 2C, in BP, the regulation of lymphocyte differentiation, ironion transport, positive regulation of tyrosine phosphorylation of STAT rotein was dominant. MF was significantly enriched in glucocorticoid eceptor binding, Wnt-activated receptor activity, ubiquitin conjugating genzyme binding. In CC, they were mainly located in recycling endosome, early endosome, endosome membrane. A KEGG enrichment analysis indicated that model-related genes were associated with the hemotopoietic cell lineage, significantly associated with the HIF-1 signaling pathway.

3Validation of a prognostic risk model for differentially expressed genes related to immune ferroptosis.

**FIGURE 3:**
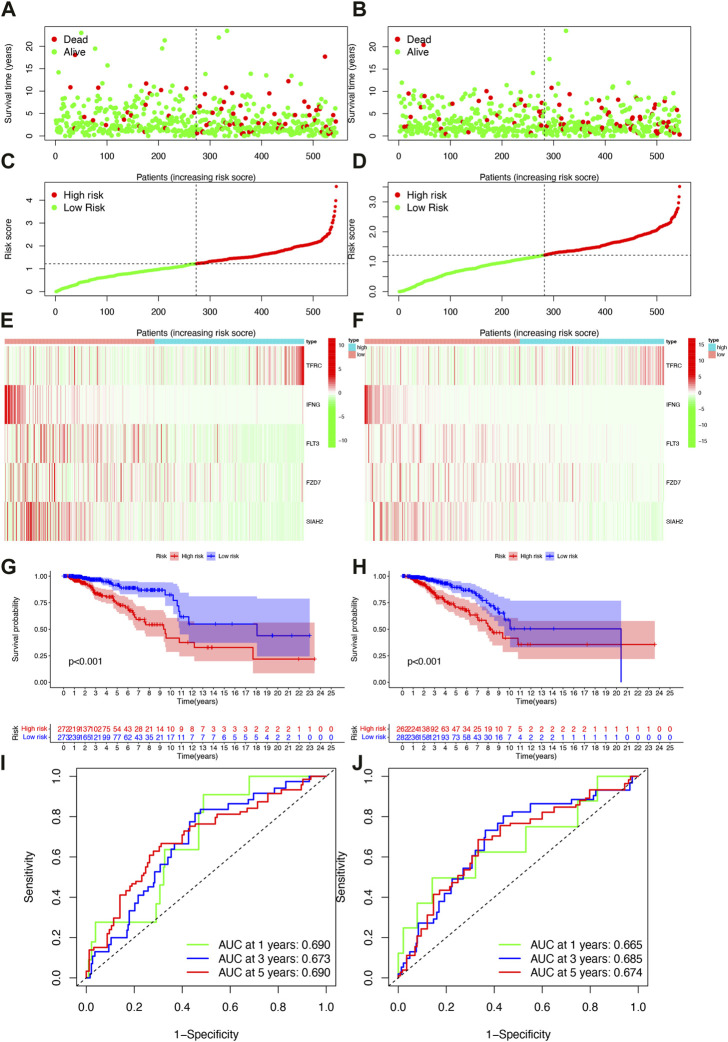
Survival analysis of patients in both the training and validation cohorts. **(A)** Distribution plots of survival times in the training cohort. **(B)** Distribution plots of survival times in the validation cohorts. **(C)** Scatter plots of risk scoresin the training cohort. **(D)** Scatter plots of risk scores in the validation cohorts. **(E)** Gene expression levels in the training cohort. **(F)** Gene expression levels in the validation cohorts. **(G)** Overall survival (OS) in the training cohort. **(H)** Overall survival (OS) in the validation cohorts. **(I)** Time-dependent ROC curves in the training cohort. **(J)** Time-dependent ROC curves in the validation cohorts.

Based on the median risk scores of the training and validation cohorts and the test cohorts, all patients were divided into high- or low-risk groups with each group accounting for 50%. As the risk score increased, so did the number of patient deaths (Figures 3A–D). The risk model-related genes expression level between high- and low-risk groups were shown in Figures 3E, F. In both trainning and validation cohorts, OS was significantly different (Figures 3G, H, *p* < 0.001). Low risk patients had better clinical outcomes than high risk patients in each cohort, which was consistent with both groups’ results. The survival time ability of IFRSig was assessed using a time-dependent ROC curve. The areas under the curve (AUC) at 1, 3, and 5 years were 0.690, 0.673, and 0.690 for the training cohort (Figure 3I) and 0.665, 0.685, and 0.674 for the validation cohort (Figure 3J), respectively. The results of all studies suggest that IFRSig can accurately predict OS.

### 3.3 Heatmap and GO/KEGG pathway enrichment analysis

**FIGURE 4:**
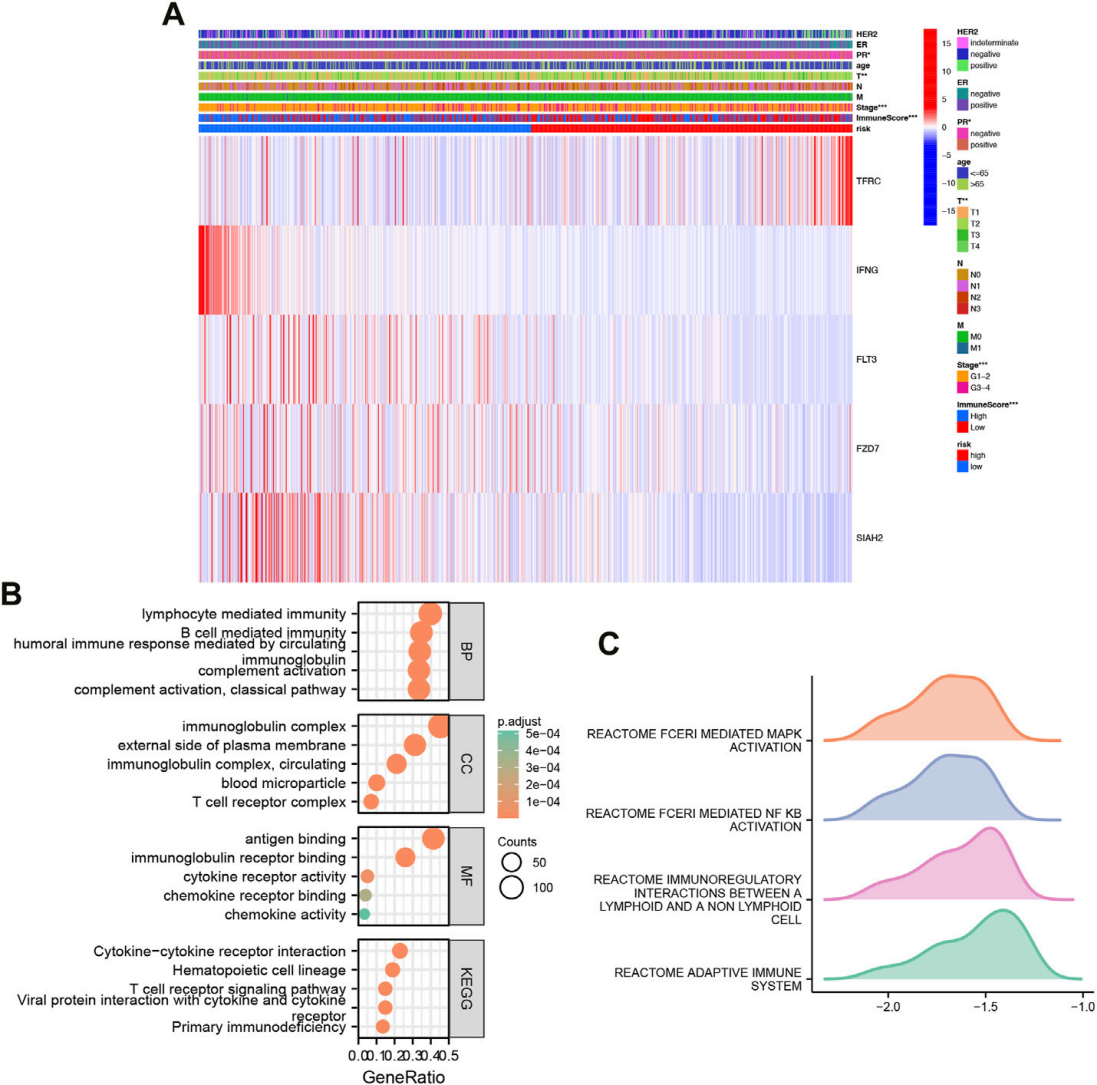
Heatmap and GO/KEGG pathway enrichment analysis. **(A)** Clinically relevant heatmap. A heatmap based on data on the clinicopathological characteristics of the patients was created based on the risk characteristics associated with prognosis. *p < 0.05, **p < 0.01, ***p < 0.001. **(B)** Gene Ontology (GO) and Kyoto Encyclopedia of Genes and Genomes (KEGG) analysis of high and low risk differential genes. **(C)** GSEA analysis of high and low risk differential genes.

Based on clinical features, we created heatmap to compare the expression relationship of prognostic model-related genes between high-risk and low-risk subgroups, and the status of HER2, ER, PR, age, T, N, M, stage, immune score were shown as patient annotations (Figure 4A).

Classification analysis revealed that GO: BP was mainly concentrated in classical pathway, humoral immune response mediated by circulating immunoglobulin, complement activation, B cell mediated immunity, lymphocyte mediated immunity; CC was mainly concentrated in immunoglobulin complex, immunoglobulin complex, circulating, external side of plasma membrane, blood microparticle, T cell receptor complex; MF was mainly concentrated in immunoglobulin receptor binding, cytokine receptor activity, chemokine receptor binding, chemokine activity. Importantly, KEGG was mainly enriched in Hematopoietic cell lineage, Primary immunodeficiency, Cytokine-Cytokine receptor interaction, Viral protein interaction with cytokine and cytokine receptor, T cell receptor signaling Pathway (Figure 4B).

Further GSEA, we found that high and low risk were mainly enriched in REACTOME_FCERI_MEDIATED_MAPK_ACTIVATION, REACTOME_FCERI_MEDIATED_NF_KB_ACTIVATION, REACTOME_IMMUNOREGULATORY_INTERACTIONS_BETWEEN_A_LYMPHOID_AND_A_NON_LYMPHOID_CELL, REACTOME_ADAPTIVE_IMMUNE_SYSTEM (Figure 4C).

### 3.4 Independent prognostic factors and nomogram construction

**FIGURE 5:**
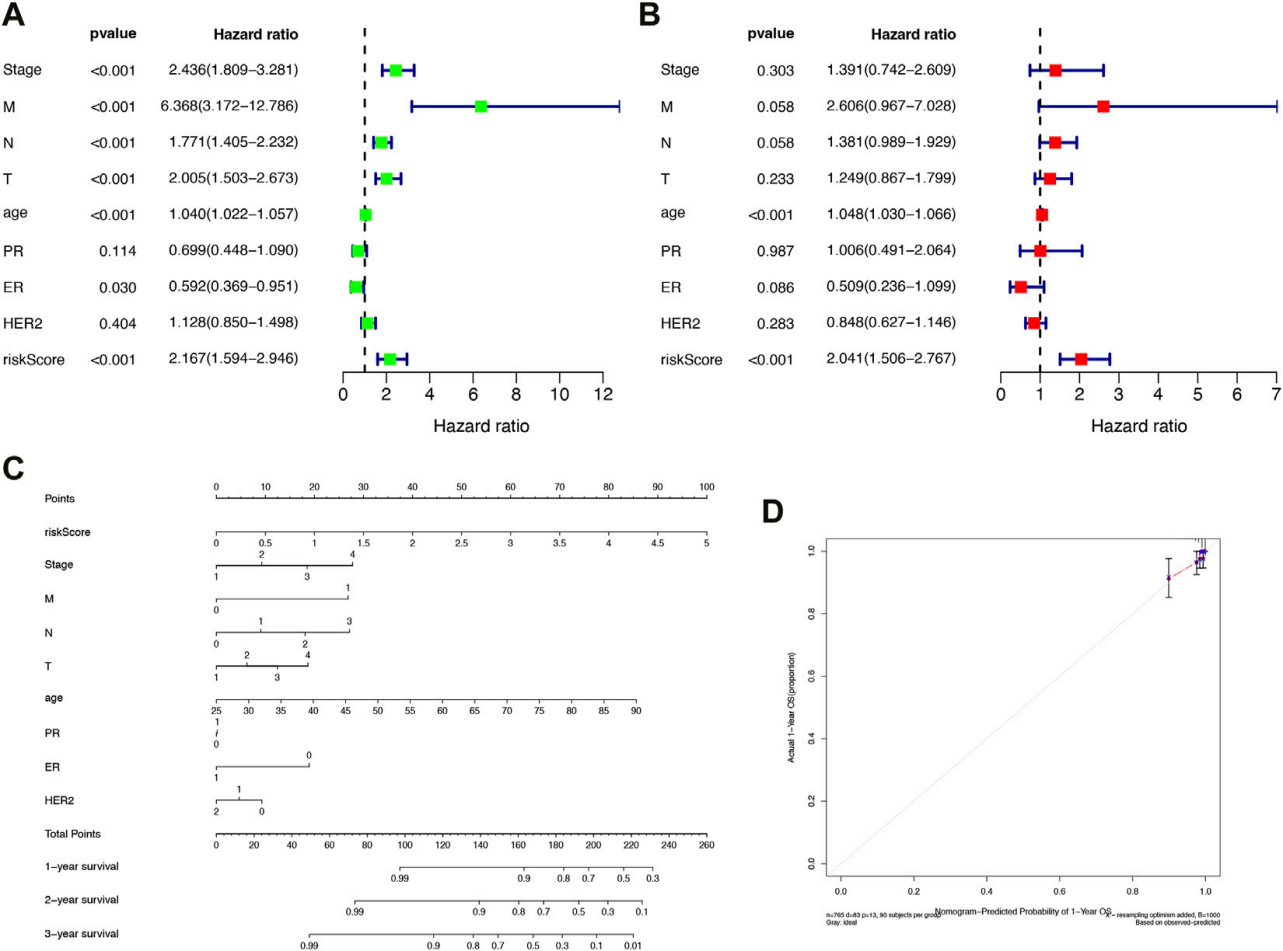
Construction of independent prognostic factors and nomogram. **(A)** Univariate Cox regression analysis. **(B)** Multivariate Cox regression analysis. **(C)** Survival nomogram based on the total TCGA cohort. **(D)** Calibration curves for predicting 1, 3, and 5-year survival of BRCA patients in the TCGA cohort. *p < 0.05, **p < 0.01, ***p < 0.001.

In the TCGA cohort, univariate Cox regression analysis showed stage, M, N, T, age, ER, and risk score were significantly associated with OS; while multivariate Cox regression analysis showed that age (*p* < 0.001) and risk score (*p* < 0.001) were significantly associated with OS (Figures 5A, B). The results showed that IFRSig was an independent prognostic factor for BRCA.

We constructed a nomogram based on risk scores and other clinicopathological covariates for calculating individualized cancer risk scores (Figure 5C). According to calibration plots, the prognostic nomogram for 1-, 3-, and 5-year OS was in agreement with the diagonal lines (Figure 5D). The outcomes demonstrated that the nomogram created by IFRSig has a good level of prognostic accuracy for BRCA patients.

## 4 Immune characteristics

**FIGURE 6:**
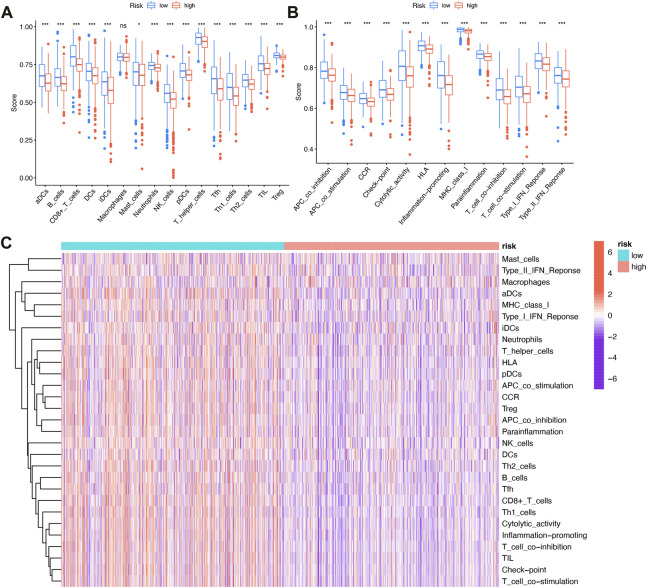
Relationship with immune infiltration **(A)** Boxplot of association between IFRSig and immune cell lineage; **(B)** Boxplot of association between IFRSig and immune function; ANOVA used as significance test, *p < 0.05, **p < 0.01, ***p < 0.001. **(C)** Immune correlation heatmap.

Tumor immune cell compositions played a major role in response to immunotherapy but the heterogeneity and dynamics of immune infiltrates in human cancer lesions remained poorly characterized. In BRCA samples, we assessed the immune infiltrating profile of immune infiltrating cells to better understand the complex crosstalk between IFRSig and immune signatures (Figure 6C). Moreover, we investigated the relationship between immune infiltrating cells and immune function as well as IFRSig, and immune infiltrating cells and immune function were found to be lower in high-risk individuals than in low-risk individuals (Figures 6A, B).

### 4.1 The immune microenvironment, immune checkpoints, immune escape

**FIGURE 7:**
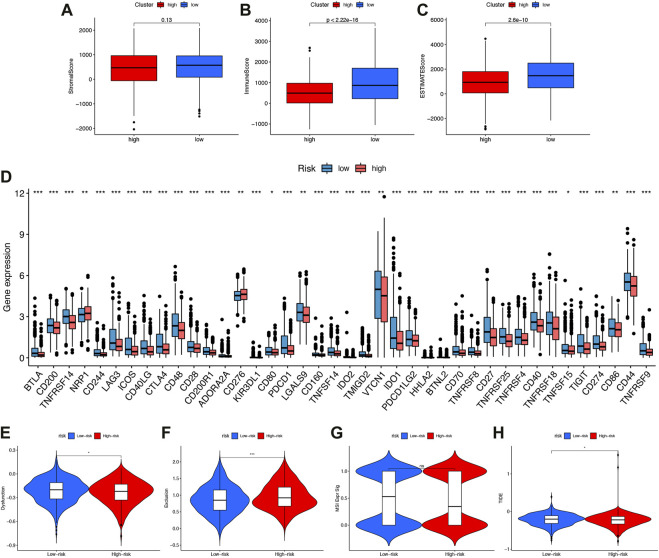
The immune microenvironment, immune checkpoints, immune escape **(A–C)** Comparison of interstitial scores, immune scores, and ESTIMATE scoresin high-risk and low-risk subgroups. **(D)** Boxplot showed association between IFRSig and immune checkpoints. *p < 0.05, **p < 0.01, ***p < 0.001. **(E–H)**Immune escape. **(E)** Dysfunction **(F)** Exclusion **(G)** MSIExprsig **(H)** TIDE score in different risk-groups.

Tumor microenvironments, as their name suggests, contained the necessary conditions for tumor cells to proliferate and metastasize. Tumor progression was influenced by immune cells, tumor cells, stromal cells, as well as a variety of active molecules. Figure 7A showed that high-risk patients have lower immune and ESTIMATE (Estimation of STromal and Immune cells in MAlignant Tumour tissues using Expression data) scores.

Our study compared the expression values of immune checkpoints molecules in patients with different IFRSigs. As shown in Figure 7B, the bar plot shows that the expression of immune checkpoints molecules were significantly lower in the high-risk score group than in the low-risk score group, except NRP1 and CD276. These findings imply that high-risk group may not benefit from anti-PD1/PD-L1/CTLA ICI immunotherapy, but from anti-NRP1/CD276.

As well, the Tumor Immune Dysfunction and Exclusion (TIDE) algorithm could predict how immune checkpoint inhibitors would react with different subgroups. Results showed that high-risk group dysfunction and TIDE scores were lower, and exclusion was higher than low-risk group exclusions (Figures 7E–H).

### 4.2 The association of immune ferroptosis-related mRNA signatures withTMB

It was reported that in many cancer types, including bread cancer, patients with higher tumor burden mutations (TMB) had lower survival rates. On the contrary, patients treated by ICI, with higher TMB generally associated with longer survival (Godenick, 1995).

**FIGURE 8:**
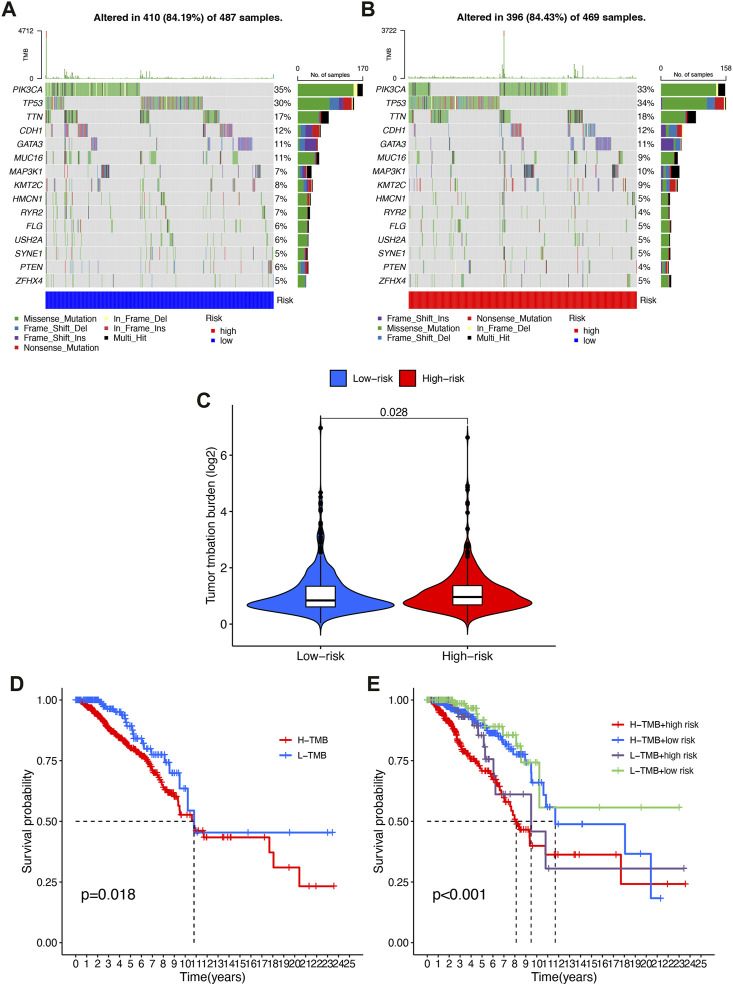
Correlation of risk score with TMB. **(A)** Oncoprint of the somatic mutational landscape of the low-risk group. **(B)** Oncoprint of the somatic mutational landscape of the high-risk group. **(C)** TMB differences between patients in low/high risk score subgroups. **(D)** Kaplan-Meier curves of high and low TMBgroups. **(E)** Kaplan-Meier Q19 curves of patients stratified by TMB and risk score.

Accordingly, we speculated that TMB might have a non-negligible relationship between prognosis risk score and TMB. Therefore, we analyzed and displayed the distribution of genetic mutations among high-risk and low-risk score subtypes. A total of 84.19% of low-risk BRCA samples had genetic mutations (Figure 8A), while 84.43% were mutated in the high-risk group (Figure 8B), indicating that samples from the high-risk group had a higher probability of gene mutation. A comprehensive landscape of somatic variation showed mutational patterns and clinical features of the top 15 most frequently changed driver genes. There were 6 genes in the low-risk group with the highest mutation frequency, including PIK3CA (35%), TP53 (59%), TTN (17%), CDH1 (12%), GATA3 (11%), MUC16 (11%). TP53 (34%), PIK3CA (33%), TTN (18%), CDH1 (12%), GATA3 (11%), MAP3K1 (10%) and other genes had the top 6 mutation frequencies in the high-risk group. A number of anticancer genes, including TP53, had a relatively high mutation rate among high-risk individuals (34% compared to 30%), MUC16 had a relatively low mutation rate in the high-risk group (9% vs 11%).

A higher level of TMB was found in the high-risk subgroup compared to the low-risk group (*p* = 0.028, Figure 8C). Patients were then assigned to different subtypes on the TMB score. There was a significant correlation between high TMB values and short overall survival (*p* = 0.018, Figure 8D). Moreover, we validated that risk score and TMB could predict BRCA prognosis without immunotherapy synergistically. As shown by the stratified survival curves, TMB status did not interfere with the risk score prognostic prediction performance. In low and high TMB status subtypes, risk score subgroups were significantly different from each other in terms of prognosis (*p* < 0.001, Figure 8E).

### 4.3 Drug sensitivity

**FIGURE 9:**
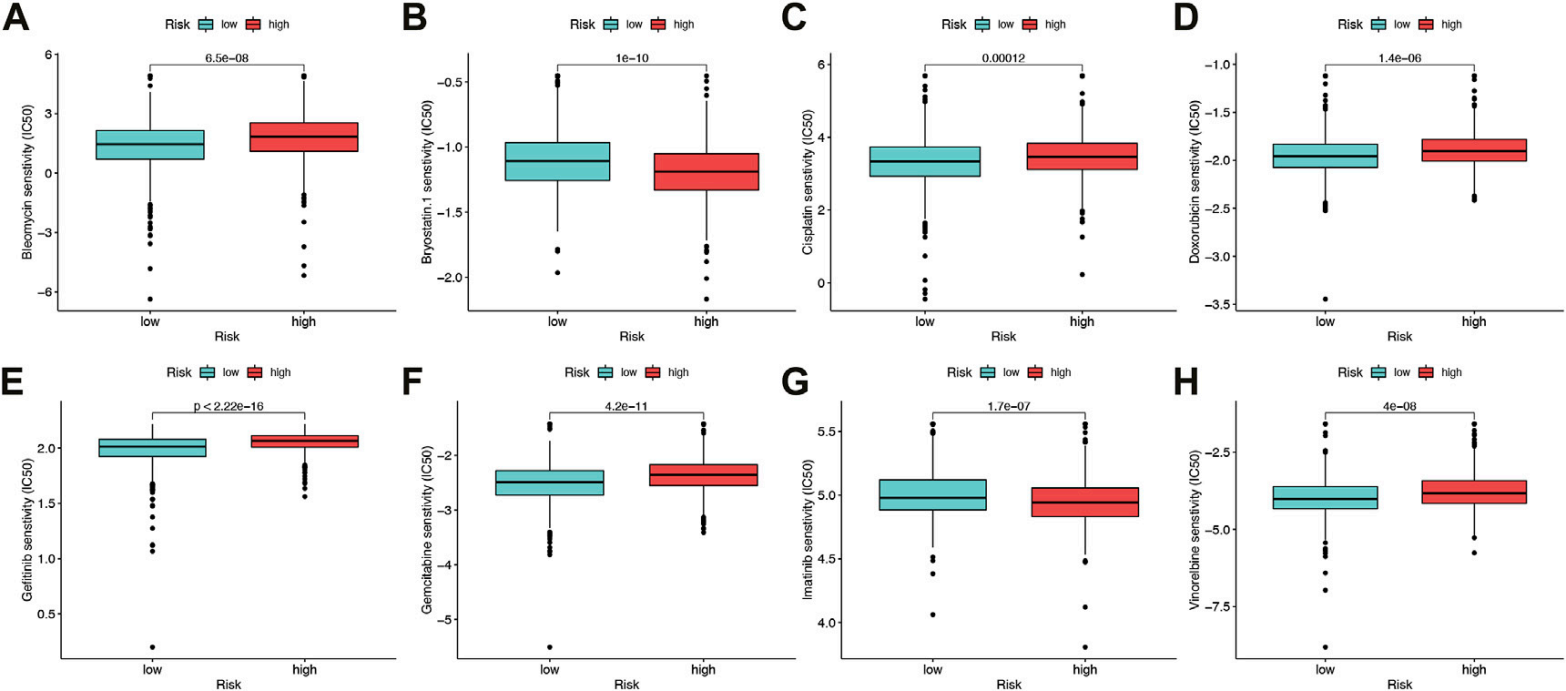
Drug sensitivity **(A–H)** Half maximal inhibitory concentration (IC50) of 8 common chemotherapeutic drugs (Bleomycin, Bryostatin, Cisplatin, Doxorubicin, Gefitinib, Gemcitabine, Imatinib, Vinorelbine). *p < 0.05, **p < 0.01, ***p < 0.001.

The sensitivity to chemotherapeutic drugs was also anticipated to better direct clinical practice because chemotherapy was a significant therapeutic approach. The IC50 of commonly used chemotherapy drugs (Bleomycin, Bryostatin, Doxorubicin, Cisplatin, Gemcitabine, Gefitinib, Imatinib, Vinorelbine) in BRCA patients in the high-risk group and low-risk group were calculated and compared by pRRophetic analysis (Figures 9A–H). In this study, it was determined that patients with a higher risk score might benefit more from chemotherapy including Bleomycin, Cisplatin, Doxorubicin, Gefitinib, Gemcitabine and Vinorelbine), while patients with a lower risk score might benefit more from chemotherapy including Bryostatin and Imatinib.

## 5 Discussion

Molecular heterogeneity, high recurrence and mortality rates, and a serious threat to women’s health make BRCA one of the most complex cancer types (Saatci et al., 2021). Early detection of BRCA is essential for effective treatment and an improved prognosis because BRCA has a poor prognosis, which has serious implications for human health and socioeconomics (Winters et al., 2017; Wang et al., 2018). Therefore, finding influential molecular markers, assessing BRCA tumor immunoreactivity, and establishing convincing prognostic models are critical for personalizing BRCA therapy.

There was a synergistic relationship between immunity and ferroptosis in tumors, according to the results of previous studies (Hong et al., 2021; Xu et al., 2021; Yang et al., 2021). In the TME, macrophages could convert from M2 to M1, making more H2O2 available for the Fenton reaction, leading to ferroptosis of tumor cells (Zanganeh et al., 2016). Another study found that activated CD8^+^ T cells release IFN- to prevent cystine from being absorbed by the body’s systems, which caused tumor cells to ferroptose through lipid peroxidation (Shao et al., 2021). When tumor cells undergo ferroptosis, tumor antigens are released, resulting in the production of immunogenic TMEs that enhance the response to immune regulation (Lu et al., 2021).

As part of this study, we performed co-expression analyses of breast cancer-related and immune ferroptosis-related genes using the TCGA. After performing a lasso regression analysis, 89 co-expressed immune ferroptosis-related DEGs were collected in order to create prognostic risk models, which could be used for both prognostic and therapeutic purposes. A high-risk and low-risk IFRSig group was created for the cancer samples. In nomograms and prognostic risk models, IFRSig was the key factor. We demonstrated a satisfactory correlation between IFRSig and clinical outcomes, indicating the IFRSig was a useful risk factor for predicting clinical outcomes. To ascertain the effectiveness of the treatment, we examined the sensitivity and resistance to chemotherapeutic drugs.

An TME consists of a complex network of tumor cells within an extremely complex internal environment formed by tumor stromal cells and their secreted active factors, as well as vascular and lymphatic networks, and extracellular matrix (Xiang et al., 2022), of which immune cells and stromal cells were the most common non-tumor cells in TME.

In addition to targeting immunogenic tumor mutations, autologous tumor-infiltrating lymphocytes (TILs) and immune checkpoint inhibitors (ICIs) could help to promote tumor growth (Bu et al., 2021; Kirtane et al., 2021), and antibodies that target PD-1, PD-L1, and CTLA-4 could be used as ICB drugs for the treatment of a variety of cancers (Han et al., 2020; Archilla-Ortega et al., 2022). Thus, we examined how risk subgroups and IC expression relate and found that high-risk patients express more NRP1 and CD276 but less CTLA-4 and PCDC1. This finding suggested that NRP1 and CD276 could be used for targeted immunotherapy for BRCA high-risk patients. Studies have shown that targeting CD276 might reduce cancer stem cell (CSC) immune escape in neck squamous cell carcinoma (HNSCC) (Wang et al., 2021). In conclusion, risk models could be employed to choose immunotherapy that was more appropriate and to forecast how well it will work for BRCA patients.

Overall, we constructed a prognostic risk signature with many advantages, but it still has some limitations. Because of tumor heterogeneity, we needed to validate our risk profile across different cohorts, and it was necessary to validate our risk profile in clinical trials. Despite the fact that our signature was still reliable because we had proven its superiority in terms of survival, tumor-infiltrating immune cells, clinicopathological features, signaling pathways, ICs, and potential small molecule drugs. Upon receiving more information and larger clinical sample sizes, our team will continue to examine and validate the risk profile.

As a result, we developed IFRSig, which was closely related to BRCA prognosis, which along with immunological features could be used to better predict clinical treatment response in patients with BRCA.

## 6 Conclusion

Our study established a prognostic risk model and identified immune ferroptosis-related genes with independent prognostic value using procedural algorithm analysis. Immune scores, immune checkpoints, and chemotherapeutic agents all showed significant correlations with prognostic models, which were then regarded as an independent prognostic feature to predict OS and clinical treatment response in BRCA patients. In this study, we gained a better understanding of how immune ferroptosis-related genes contribute to BRCA occurrence and progression.

## Data Availability

The datasets presented in this study can be found in online repositories. The names of the repository/repositories and accession number(s) can be found in the article/Supplementary Material.
